# Supplementary material for: The Antioxidant Content of Coffee and Its In Vitro Activity as an Effect of Its Production Method and Roasting and Brewing Time
Source: Antioxidants (Basel). 2020 Apr 10;9(4):308. doi: 10.3390/antiox9040308 (PMC7222172; doi:10.3390/antiox9040308)
Supplement: Supplementary file 1 [file antioxidants-09-00308-s001.pdf]

Table S1. The weather condition in time of coffee cultivation in two experimental production plantations in 2019.

country	Peru							Poland		
coffee growing stage or production stage	growing time	flower setting	blossom	harvest	harvest	drying coffee beans	packing	transport	wholesale	sale of green coffee	roasting
weather factors/month	I	II	III	IV	V	VI	VII	VIII	IX	X	XI
Cajamarca											
min. temp. (in ºC)	7.4	6.7	6.7	6.6	4.5	2.6	2.7	3.6	4.6	6.3	5.5
max. temp. (in ºC)	21.1	20.2	20.3	20.2	21.0	21.1	21.1	21.0	21.0	21.0	21.2
rainfall in mm	98.0	111.0	133.0	91.0	42.0	15.0	8.0	14.0	40.0	92.0	68.0
sun hour per month	381.3	341.6	375.1	360.0	355.9	342.0	355.0	357.1	360.0	376.7	367.5
humidity (in %)	65.0	61.0	68.0	66.0	60.0	58.0	57.0	57.0	60.0	62.0	62.0
Mendoza	I	II	III	IV	V	VI	VII	VIII	IX	X	XI
min. temp. (in ºC)	16.0	15.0	14.0	12.0	5.0	3.0	2.8	3.0	5.0	7.2	10.0
max. temp. (in ºC)	32.0	28.0	26.0	20.0	19.0	17.2	16.8	15.3	20.0	22.0	24.0
rainfall in mm	35.0	32.0	25.0	12.0	8.0	5.0	9.0	4.0	9.0	11.0	17.0
sun hour per day	452.6	383.6	378.5	335.7	318.4	287.7	313.1	326.1	345.0	387.8	404.4
humidity (in %)	60.0	58.0	62.0	63.0	62.0	61.0	58.0	57.0	56.0	55.0	54.0
